# Incompatibility and Competitive Exclusion of Genomic Segments between Sibling *Drosophila* Species

**DOI:** 10.1371/journal.pgen.1002795

**Published:** 2012-06-28

**Authors:** Shu Fang, Roman Yukilevich, Ying Chen, David A. Turissini, Kai Zeng, Ian A. Boussy, Chung-I. Wu

**Affiliations:** 1 Biodiversity Research Center, Academia Sinica, Taipei, Taiwan, Republic of China; 2 Department of Ecology and Evolution, University of Chicago, Chicago, Illinois, United States of America; 3 Biology Department, Union College, Schenectady, New York, United States of America; 4 Department of Biology, Loyola University Chicago, Chicago, Illinois, United States of America; 5 State Key Laboratory of Biocontrol, School of Life Sciences, Sun Yat-sen (Zhongshan) University, Guangzhou, People's Republic of China; 6 Beijing Institute of Genomics, Chinese Academy of Sciences, Beijing, People's Republic of China; University of Washington, United States of America

## Abstract

The extent and nature of genetic incompatibilities between incipient races and sibling species is of fundamental importance to our view of speciation. However, with the exception of hybrid inviability and sterility factors, little is known about the extent of other, more subtle genetic incompatibilities between incipient species. Here we experimentally demonstrate the prevalence of such genetic incompatibilities between two young allopatric sibling species, *Drosophila simulans* and *D. sechellia*. Our experiments took advantage of 12 introgression lines that carried random introgressed *D. sechellia* segments in different parts of the *D. simulans* genome. First, we found that these introgression lines did not show any measurable sterility or inviability effects. To study if these *sechellia* introgressions in a *simulans* background contained other fitness consequences, we competed and genetically tracked the marked alleles within each introgression against the wild-type alleles for 20 generations. Strikingly, all marked *D. sechellia* introgression alleles rapidly decreased in frequency in only 6 to 7 generations. We then developed computer simulations to model our competition results. These simulations indicated that selection against *D. sechellia* introgression alleles was high (average *s* = 0.43) and that the marker alleles and the incompatible alleles did not separate in 78% of the introgressions. The latter result likely implies that most introgressions contain multiple genetic incompatibilities. Thus, this study reveals that, even at early stages of speciation, many parts of the genome diverge to a point where introducing foreign elements has detrimental fitness consequences, but which cannot be seen using standard sterility and inviability assays.

## Author Summary

Determining the extent of genomic incompatibilities is a pivotal issue in understanding the process of speciation. A controversial topic that has recently sparked debate is whether there are few isolated genetic regions (so-called “genomic islands of speciation”) or extensive genetic regions (“genomic continents of speciation”) responsible for species divergence. To answer this question, most work has focused on species divergence at the DNA sequence level. Here, we present a new perspective by shifting the focus to the fitness and functional aspects of foreign genomic introgression. To illustrate our point, we performed an introgression experiment on two sibling species, *D. sechellia* and *D. simulans*. After introgressing random genomic segments of *D. sechellia* into *D. simulans* genetic background, a 20-generation competition experiment revealed that, even at the early stages of speciation, there are virtually always detrimental fitness consequences to introducing random foreign elements from one genome to another. This implies that incipient speciation may be characterized by widespread accumulation of genomic incompatibilities rather than a few isolated genes. This study shows that we should move beyond the sterility and inviability assays in order to understand the full extent of genetic incompatibilities during speciation.

## Introduction

Explaining the present-day biological diversity requires an understanding of the speciation process. While we typically cannot observe speciation, we can ask to what extent are species incompatible if brought together to form hybrids. The founders of the Modern Synthesis typically argued that even the most recently diverged species accumulate enough fitness differences such that no large part of the genome can be shared between them (e.g. [1]–[3]). This view of speciation argues that lots of loci with a wide range of effects on fitness should characterize the speciation process. E. Mayr championed a “genetic revolutions” version of this view, arguing that once separated from gene flow, most of the genome will undergo rapid coadaptive change, resulting in widespread fitness differences during speciation [3], [4]. As a result, the Biological Species Concept (BSC) has historically emphasized the cohesiveness of the species, where most of the genome diverges as a single biological unit and the evolution of isolating barriers play a central role in protecting its “integrity” [2], [3], [5]. On the other hand, if adaptive functional divergence involves a limited number of loci, much of the genome could still penetrate across the species boundary during incipient stages of speciation. This is often described as the “genic view” of speciation and is argued to be especially applicable when speciation occurs with gene flow (i.e. parapatric and sympatric modes of speciation, [6]–[8]; see Figure 1 in [6]).

**Figure 1:**
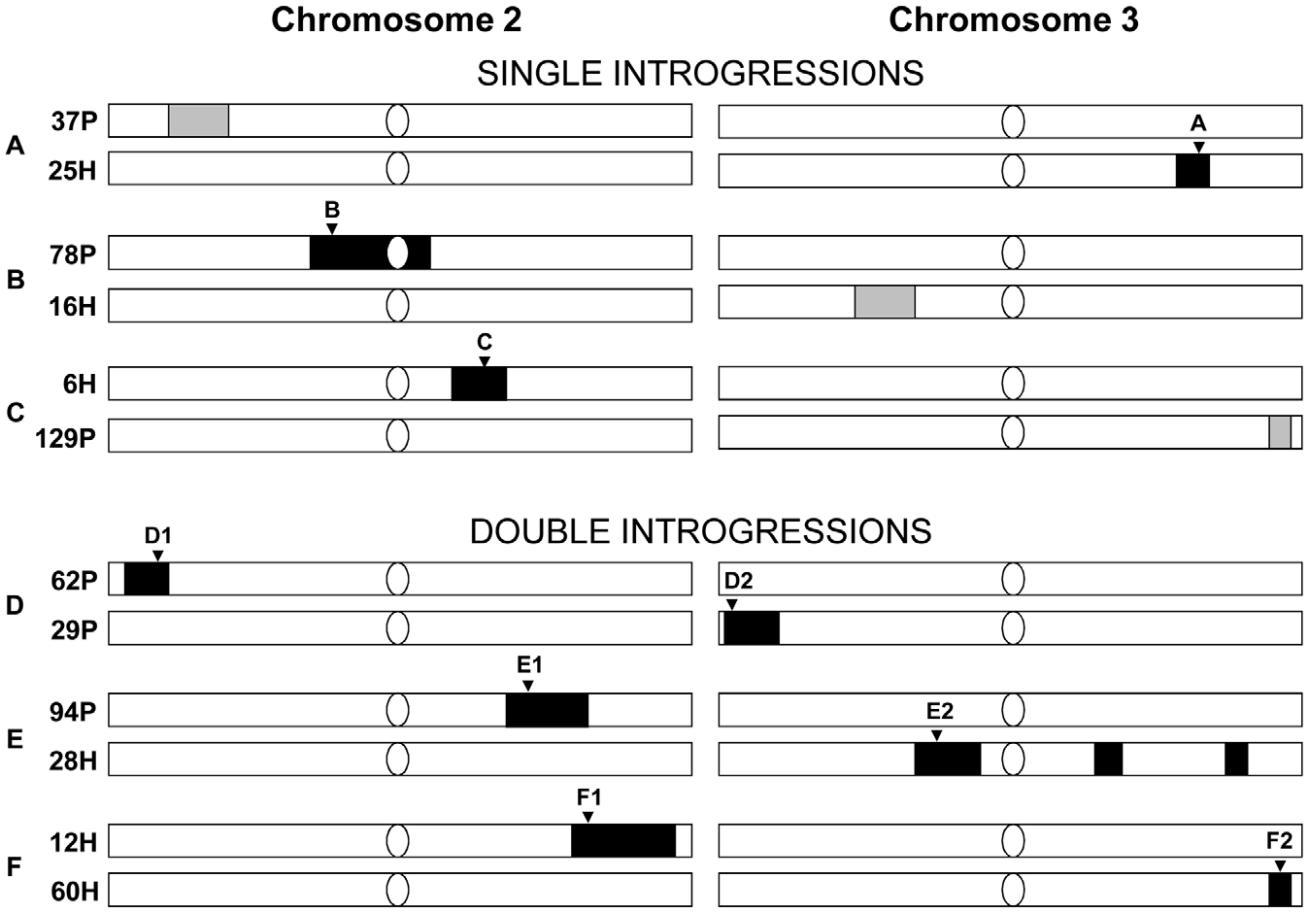
The introgression lines for establishing initial generation of six cross sets for competition experiments. The light bars represent *simulans* chromosome II or III, and the dark blocks represent *sechellia* introgression regions. Confirmed introgressions are shown in black. Unconfirmed introgressions are shown in grey. Microsatellite markers tracked in the experiment are labeled by the inverted triangles: A: DMU25686 (cytological position: 93F14); B: DRODORSAL (36C8); C: DROGPAD (47A9); D1: AC005732 (cytological position 24C9); D2: DMRHO (62A2); E1: DMMP20 (49F13); E2: DMCATHPO (75E1); F1: DS00361 (54B5); F2: DMU43090 (99D5) [53], [54] (see Materials and Methods for details of each marker and for single versus double introgression designs).

Recently, several studies have attempted to look for the so-called “genomic islands of speciation” (e.g. [9]–[12], see review in [13]). These assume that speciation with gene flow has occurred and that it will homogenize the genome except for a few genes involved in reproductive isolation and differential adaptation [6], [8], [14]. While earlier studies found support for the “islands” of speciation (e.g. [9]–[11]), more recent comprehensive genome-wide screens are revealing a different picture [15]–[17]. Rather than having small genomic islands surrounded by mostly undifferentiated genomes, these incipient and sympatric races show widespread genomic differentiation, either being randomly distributed across the genome or clustered in the so-called “genomic continents” such as inversions or particular chromosomes (see [15] for discussion).

Other studies focus on identifying “speciation genes” that underlie reproductive isolation between closely related species (see [5] for review). Historically, these studies have been interested in determining how many loci are involved in reproductive isolation [18]–[23], and elucidating their identity and their evolution [24]–[31]. The great majority of these studies focus on the more easily measurable effects of sterility and inviability of hybrids. Many such sterility and inviability factors differentiating closely related species have been identified (see reviews in [5], pg. 302; [32]).

While both approaches have made important contributions to understanding the genetics of speciation in nature, neither addresses the degree to which two genomes are genetically incompatible. Genome-wide scans show us the extent of sequence divergence across whole genomes, but they say nothing about whether these divergent sites carry fitness or functional consequences. Studies that search for speciation genes concentrate a priori on such effects as hybrid sterility and inviability, but ignore the rest of the genome for other fitness and functional differences between species. Perhaps genetic studies of natural hybrid zones and hybrid fitness come closest to estimating the true extent of genetic incompatibilities between incipient species (e.g. [33], [34]). Results from hybrid zones suggest that many fitness-related genes may differentiate genomes of even incipient races or recently diverged sibling species [33], [35], [36]. However, little has been done to determine whether these incompatibilities are associated with sterility or inviability effects or contain other fitness detriments. Further, the hybrid studies cannot identify specific genomic regions responsible for incompatibilities or determine the strength of selection associated with each of these genetic incompatibilities. Exploring these questions in a laboratory setting using genetic introgressions provides the best means to estimate the basic parameters of genetic incompatibilities on a genome-wide level.

To approach this general question, the present paper focuses on recently diverged sibling species *Drosophila simulans* and *D. sechellia*. Molecular evidence indicates that they have diverged only about 250,000 years ago and thus represent fairly early stages of speciation [37]. For instance, these species have accumulated partial, but incomplete premating isolation and still produce fertile hybrid females in F_1_ and subsequent generations [18], [38]. These sibling species have most likely speciated allopatrically; *D. simulans* likely evolving on the African continent, while *D. sechellia* has remained an island endemic to the Seychelles archipelago in the Indian Ocean [39], [40]. Today, both species can be found in the Seychelles archipelago, but seem to occupy different islands [39]. Thus, we address our main question about genome-wide incompatibilities in a relatively young pair of taxa where whole-genomes were likely able to diverge without being impeded by substantial gene flow.

To determine the extent and nature of genetic incompatibilities between *D. simulans* and *D. sechellia*, we have introgressed random genetic segments from *D. sechellia* into a *D. simulans* genome. We first ask if these random introgressions contain measurable sterility and/or inviability effects. If some of these introgressions do not show sterility or inviability, we can then ask whether these regions are selectively neutral upon introgression or whether they carry other deleterious fitness effects after long-term genetic competition experiments. If these random genomic introgressions turn out to be selectively neutral, this would indicate that genomic incompatibilities are typically restricted to previously described genes associated with such effects as sterility and inviability (e.g. see [32]). However, if we find that most introgressions placed into a foreign genetic background experience strong fitness reduction and are selected out of the host population, it would imply that we are fundamentally underestimating the extent and possibly the type of fitness differences that accumulate between species. Thus our paper highlights the need to incorporate competition and other selection experiments to accurately test theories related to “genomic islands of speciation”.

## Results

### No detectable fertility differences between introgression lines and the wild-type *D. simulans* line

The present study utilized 12 recombinant introgression lines (RILs; henceforth referred to as “introgression lines” for short) from Stuart J. Macdonald, Isabel Colson and David B. Goldstein (Oxford University). Briefly, each line was made by genetically introgressing *D. sechellia* chromosomal fragments into a *D. simulans* genetic background (for a detailed description of the construction of these lines see *Materials and Methods*). The introgressions were made homozygous by single-pair sib-mating for 18 generations, and 41 microsatellite genetic markers across X, 2nd, and 3rd chromosomes were used to map the regions of the *sechellia* introgressions. As those introgression lines have been maintained in Goldstein's laboratory for several years, we therefore tested whether each line was homozygous for the expected *sechellia* introgression (henceforth referred to as “confirmed lines”) or whether it did not contain the *sechellia* marker allele (henceforth referred to as “unconfirmed lines”; see Figure S1). The latter lines may have lost the introgression by stochastic or other processes during their years of maintenance. In total, 9 lines were confirmed to carry *sechellia* introgressions and 3 lines failed to show introgressions.

To test whether the created introgression lines had any obvious inviability and/or sterility factors, we assayed overall fertility of each introgression line and compared it to the fertility of the experimental *simulans* strain that was used as the genetic background of introgressions (Table 1). Our results showed that while the introgression experiment clearly increased the variance in fertility among introgression lines (one-way ANOVA: *F* = 9.05, *d.f.* = 11, *p*<0.0001), the average fertility among lines was nearly identical to that of the experimental *simulans* strain (Table 1). Further, there was no evidence of significant fertility reduction in any of the lines studied using the posthoc Tukey-Kramer HSD test (Table 1). There was also no trend in fertility reduction among our introgression lines, with five out of the eight tested introgression lines actually having higher fertility than the experimental *simulans* strain (Table 1). Similarly, crosses between different introgression lines and between their F_1_ progeny either resulted in non-significant differences in fertility from the *simulans* strain or higher fertility relative to the *simulans* strain (see Tables S2 and S3). Therefore, we conclude that the present *sechellia* alleles placed in a *simulans* genetic background did not generate any detectable inviability and/or sterility effects. We can begin to address our main question as to whether these *sechellia* introgressions are equally fit to wild-type *simulans* alleles in a *simulans* genetic background.

**Table 1:** Fertility (viability+fecundity) assays of introgression lines and the *simulans* strain.

Lines	Progeny Mean ± SE	Tukey-Kramer HSD test value*	Significance
sim 132 (*D. simulans*)	289±82.4		NS
introgressions (average)	291		
25H	-	-	-
78P	324±74.8	−62.894	NS
6H	313±58.8	−73.994	NS
62P	334±76.3	−53.494	NS
29P	329±39.7	−57.694	NS
94P	214±30.8	−22.294	NS
28H	211±65.6	−19.294	NS
12H	263±84.8	−71.494	NS
60H	339±36.4	−48.294	NS

Note: Fertility was measured by the total progeny produced by three pairs of flies for 15 days (N = 10).

*: Tukey-Kramer HSD value here shows significant difference between the sim132 *D. simulans* strain and introgression strains, taking into account multiple testing. It is equal to Abs(Mean[i]-Mean[j])-LSD. The value must be positive to be significant at *P-value*<0.05. NS = not significant. All analyses were performed using JMP software (SAS). 25H line was lost before it could be tested.

### Competition tests between introgressions and wild-type segments reveal repeated declines of *D. sechellia* alleles

To test whether the introgressions had any other deleterious fitness affects, we set up 6 independent competition experiments to determine the evolutionary fate of *sechellia* alleles in a *simulans* genetic background. For each competition experiment, we crossed two different introgression lines (see Methods for details). Combining the introgression lines together allowed us to control for any non-intentional effects of the introgression procedure (e.g. to control for different levels of inbreeding). Competition crosses were of two types: The first set of experiments crossed a line containing a single confirmed introgression with another line that did not show evidence of the introgression. Thus in this experiment, *sechellia* alleles were competing with the wild-type *simulans* alleles at only a single genomic region (henceforth referred to as “single-introgression experiment”; shown in Figure 1A–1C as black blocks). The second type of experiment crossed two lines, each containing a unique confirmed introgressed region on either the 2^nd^ or 3^rd^ chromosome (henceforth referred to as “double-introgression experiment”; Figure 1D–1F). This allowed us to see if the introgressions on the 2^nd^ and 3^rd^ chromosomes interact when each competed against the wild-type *simulans* alleles (see below).

**Figure 2:**
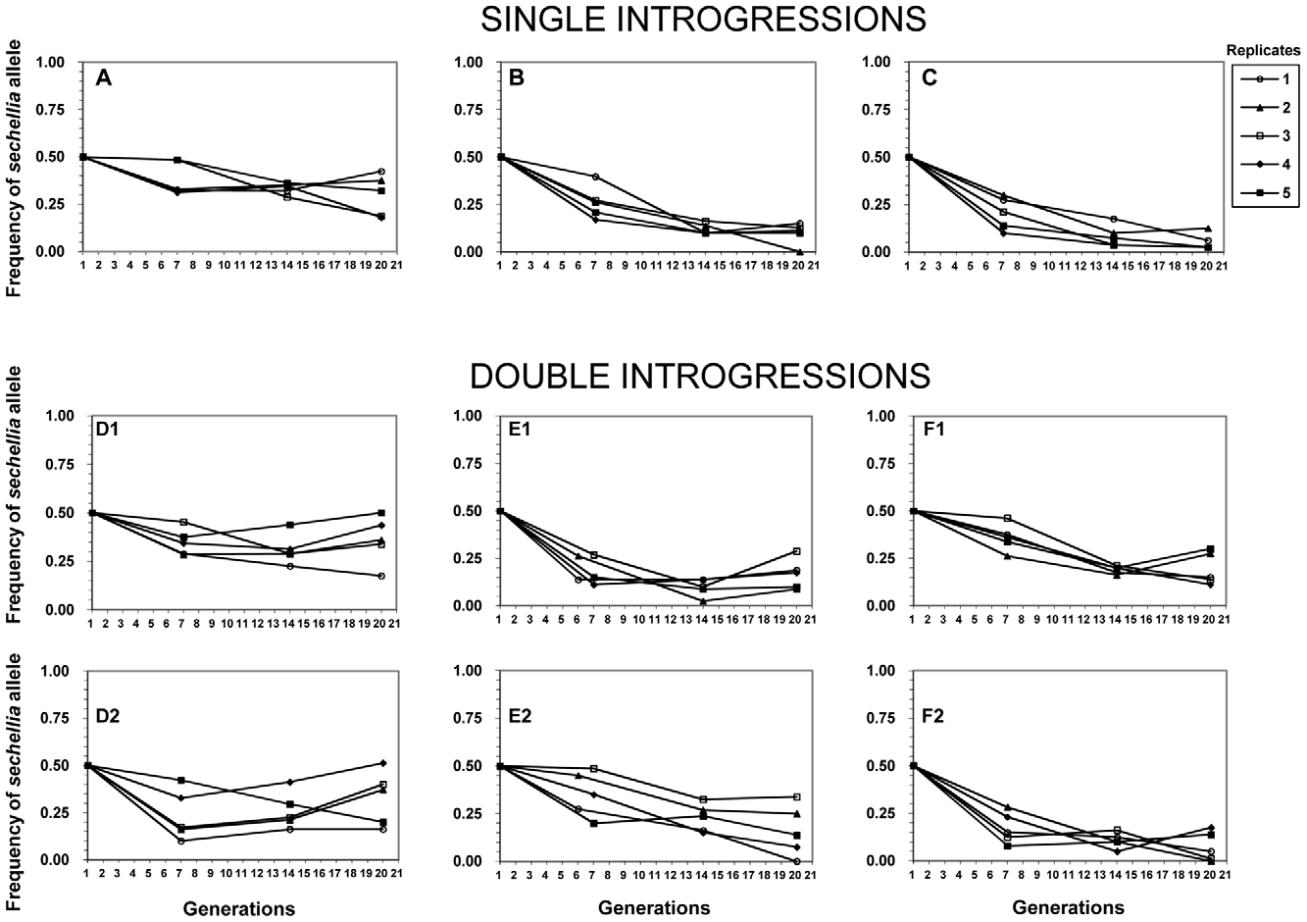
Frequencies of the nine *sechellia* markers in the six competition experiments over 20 generations. X-axis: generations. Y-axis: frequencies of *sechellia* markers. Each plot monitors the frequency of a single *sechellia* introgression relative to its *simulans* counterpart. A–C plots represent experiments with only a single introgression per genotype, while D1–F2 plots represent experiments with two *sechellia* introgressions per genotype, even though each plot follows only a single introgression/marker. Five experimental replicates for each marker. See Figure S1 for introgression details.

Our competition experiments revealed highly unexpected results based on the above lack of difference in fertility between introgression lines and the wild-type *simulans* line. We found that all of the *sechellia* marker alleles sharply decreased in their frequencies relative to the wild-type *simulans* alleles by generations six and seven (Figure 2). We found that from the starting 50% frequency, the *sechellia* marker alleles dropped to a range of 38% to 17% among different experiments. After this initial drop, the frequencies of *sechellia* alleles either: 1) kept further declining, 2) remained relatively unchanged or 3) actually increased over time in subsequent generations. This striking observation resulted in several conclusions. First, it showed that introgressed segments of *sechellia* into a *simulans* background does indeed carry strong deleterious fitness consequences. Second, it showed that these fitness effects cannot be detected by standard fertility measures above and were only revealed through long-term competition experiments. Third, it indicated that the marker alleles we were tracking either remained genetically linked to the deleterious alleles at surrounding fitness loci or became independent over time from these deleterious alleles due to recombination.

### No detectable fitness epistasis between introgressed *D. sechellia* segments in a *D. simulans* background

We then tested whether the frequency declines of *sechellia* alleles are affected by having one or two confirmed introgressions during the competition experiment (i.e. single introgressions versus double introgressions). Because the two *sechellia* introgressed segments are on different chromosomes, these are expected to assort independently during competition. We found that particular *sechellia* marker alleles that were either in the presence of a single or a double introgression had nearly identical frequency declines after six or seven generations (t test: single avg. freq._gen.6/7_ = 0.284, double avg. freq. _gen.6/7_ = 0.276; *F* = 0.048, *p* = 0.866; see also Figure 2). Similarly, after 20 generations of competition, both types of introgression designs showed very similar *sechellia* marker frequencies (t test: single avg. freq._gen.20_ = 0.158, double avg. freq. _gen.20_ = 0.214; *F* = 1.58, *p* = 0.216; Figure 2). Thus we did not detect any significant differences between single and double introgressions on fitness.

Finally, we tested whether there is linkage disequilibrium between 2^nd^ and 3^rd^ chromosome marker alleles in double introgression experiments (experiments D, E, and F in Figure 1). Except for few cases in experiment D, experimental populations did not deviate significantly from linkage equilibrium (*p*>0.05; Table S1). Thus using this approach we failed to detect evidence for epistasis between 2^nd^ and 3^rd^ chromosome *sechellia* introgressions. In total, these results suggest that the observed fitness reduction during competition is not a consequence of combining two introgressions on 2^nd^ and 3^rd^ chromosome together. This implies that each *sechellia* segment is negatively epistatically interacting with the *simulans* genetic background on its own.

### Computer simulations suggest that multiple incompatibilities exist within each introgressed segment

To estimate the intensity of selection against *sechellia* alleles and the recombination rate between the marker and the surrounding fitness loci, we performed multiple-generation, computer simulations using maximum likelihood approaches. We assumed that each microsatellite marker is neutral and is linked at a recombination distance of *c* to a single deleterious allele with selection coefficient *s* and dominance *h*. All other aspects of the competition experiment, such as experimental population sizes, recombination only in females, etc., were simulated accordingly (see *Materials and Methods* for details). Because our main interest is to estimate *c* (recombination rate; ranging from 0 to 0.5) and *s* (selection against *sechellia* allele; ranging from 0 to 1), we manipulated the dominance parameter, *h*. These estimates were not meant to precisely estimate *s* and *c*, but to give an idea of the scale of these values necessary to produce the observed marker allele frequency declines. The *h* parameter was assumed to equal either: 0, 0.5, 0.9 or 1. Thus, we allowed *sechellia* allele to become increasingly dominant over the *simulans* allele from complete recessivity (*h* = 0) to complete dominance (*h* = 1). In reality, it is not known which dominance best characterizes the *sechellia*-*simulans* allelic relationship, but as we will see below, our results are robust to changes in the dominance parameter.

Maximum likelihood estimates of *s* and *c* were obtained by comparing the observed *D. sechellia* marker frequencies to computer-generated distributions based on simulations of introgression lines. Table 2 summarizes the simulation results based on contour plots in Figure S2. Interestingly, we found that 7 of the 9 (78%) maximum likelihood estimates of *c* have values that are very close to 0, corresponding to very small physical distances (Table 2). This result has two possible interpretations: First, it may imply that the marker and fitness locus happen to be very close to each other in 78% of the experiments. Second, rather than a single fitness locus per segment (as our simulation model assumed), the introgressed segments may be carrying multiple fitness loci with deleterious effects, thus preventing the single marker from recombining away from multiple deleterious interactions. Given that our markers were chosen randomly and that the *sechellia* segments are fairly large (Figure 1, Figure S1), the chance that each randomly chosen marker locus happened to be so close to a single fitness locus with a deleterious effect seems very low. Instead these results most likely suggest that the introgressed *sechellia* segments probably carry multiple deleterious fitness alleles in a *simulans* background. Table 2 also shows that varying the dominance parameter, *h*, does not change the major results of the simulations, with essentially presence or absence of positive recombination across different lines.

**Table 2:** Summary of maximum likelihood estimates of recombination rate (*c*) and selection coefficient (*s*) parameters with different dominance (*h*).

Introgression segments	Recombination rate (*c*)					Selection coefficients (*s*)				
	*h* = 0	*h* = 0.5	*h* = 0.9	*h* = 1	Average	*h* = 0	*h* = 0.5	*h* = 0.9	*h* = 1	Average
A	0.1	0.2	0.4	0.3	0.25	0.7	0.5	0.5	0.4	0.525
B	0	0	0	0	0	0.5	0.3	0.2	0.2	0.30
C	0	0	0	0	0	0.9	0.4	0.3	0.2	0.45
D1	0.2	0.5	0.2	0.2	0.275	0.9	0.9	0.4	0.4	0.65
D2	0	0	0	0	0	0.2	0.4	0.3	0.6	0.38
E1	0	0	0	0	0	0.5	0.6	0.5	0.5	0.53
E2	0	0	0	0	0	0.3	0.2	0.3	0.3	0.28
F1	0	0	0	0	0	0.3	0.2	0.5	0.5	0.38
F2	0	0	0	0	0	0.9	0.3	0.3	0.2	0.425
Average	0.03	0.08	0.07	0.06	0.06	0.58	0.42	0.37	0.37	0.43

Note: Data was summarized from contour plots described in Figure S2.

Finally, our computer simulations revealed that selection coefficients against *sechellia* alleles must be strong in order to explain the observed evolutionary changes (Table 2). On average, the selection intensity against *sechellia* alleles was *s* = 0.43 with a range of 0.28 to 0.65. It can also be seen that the estimated selection coefficients were negatively correlated with the dominance of *sechellia* alleles (*R^2^* = 0.89, *F* = 27.4, *p* = 0.034). This result is in general agreement with expectation of Haldane' sieve [41], since if alleles are more recessive, in order to explain the observed frequency declines, they must have stronger selection coefficients (note however that we are dealing with negative selection rather than positive as in [41]). However, even under completely dominant assumption, the selection strength against *sechellia* alleles is on average still high (*s* = 0.37; Table 2). In total, our simulations indicated that multiple incompatibilities likely exist within the great majority of our introgressed segments and that these factors have substantial negative fitness consequences that cannot be detected by standard fertility tests above.

### Weak evidence for reduced mating success is responsible for declines of *D. sechellia* alleles from *D. simulans* genetic background

Determining exactly why *sechellia* alleles declined in frequency in our competition experiments is beyond the scope of this paper. However, we did perform one additional experiment focusing on whether introgression lines have reduced mating success relative to the original *simulans* strain (see *Materials and Methods* for details). These results showed that individuals (combined males and females) from 6 out of 8 (75%) introgression lines did indeed have lower relative mating success compared to individuals from the *simulans* strain (Table 3). While suggestive, this result is not statistically significant (sign test: one-tailed *p* = 0.14). On average, *simulans* individuals comprised 53% of the total matings relative to 47% of the introgression individuals, which did turn out to be slightly significant (Wilcoxon test: χ^2^ = 6.4, *p*<0.011). It is particularly the introgression males that are strongly outcompeted by *simulans* males (a 12% differential in fitness; Wilcoxon test: χ^2^ = 10.6, *p*<0.0011). Introgression females have the same mating success as *simulans* females (Wilcoxon test: χ^2^ = 0.03, *p* = 0.87; Table 3). Unfortunately, performing such an experiment does not allow us to adequately control for different overall levels of inbreeding between our introgression lines and our *simulans* line, a factor known to influence mating behavior in *Drosophila* (e.g. [42]). Thus, presently, we cannot conclude that mating behavior differences were responsible for the observed inferiority of *sechellia* alleles in a *simulans* background (see Discussion for additional possibilities).

**Table 3:** Mating success tests between each introgression line and the *simulans* strain.

Lines	# of cage replicates	N_SS_	N_SI_	N_IS_	N_II_	N_Total_	Introgression %	Introgression ♀ %	Introgression ♂ %
25H (A)	-	-	-	-	-	-	-	-	-
78P (B)	2	38	19	34	29	120	0.46	0.53	0.40
6H (C)	3	44	39	40	23	146	0.43	0.43	0.42
62P (D1)	2	32	32	27	29	120	0.49	0.47	0.51
29P (D2)	2	24	20	46	30	120	0.52	0.63	0.42
94P (E1)	2	47	30	18	23	118	0.40	0.35	0.45
28H (E2)	4	58	46	74	61	239	0.51	0.56	0.45
12H (F1)	2	41	18	32	29	120	0.45	0.51	0.39
60H (F2)	3	47	38	52	43	180	0.49	0.53	0.45
**Average introgression**		**41.4**	**30.3**	**40.4**	**33.4**	**-**	**0.47**	**0.50**	**0.44**

Note: Labels inside parentheses for each introgression line indicate the experiment performed in Figure 1. N_SS_ = *simulans* homotypic pairs, N_SI_ = *simulans* females x introgression males, N_IS_ = introgression females x *simulans* males, N_II_ = introgression homotypic pairs. 25H line (corresponding to experiment A in Figure 1) was lost before it could be tested.

## Discussion

The “genic view” of speciation typically states that genomic introgression may readily occur except for rare reproductive isolation genes (see Figure 1 in [6]). However, E. Mayr and other founders of the Modern Synthesis typically viewed genomes of different species as tightly cohesive units that become largely impenetrable to gene flow during and after speciation events (see [2], [3], [5]). Recent array and whole-genome sequencing technologies are revealing that even between incipient races, nucleotide sequence divergence is often extensive across genomes [15], [16], [17]. However, to unambiguously determine which view of speciation is closer to reality, one needs to study genome-wide genetic incompatibilities between different races and species. To approach this seminal question, we performed an introgression study in order to assess genomic fitness divergence between relatively young and most likely allopatrically diverged sibling species *Drosophila sechellia* and *D. simulans*. Our paper for the first time demonstrates that genome-wide genetic incompatibilities between young sibling species are already fairly extensive. We found that all of our 9 random introgressed genetic segments from *sechellia* into a *simulans* genome carried negative fitness consequences when competed for multiple generations against wild-type *simulans* alleles. While some of the marker alleles were able to partially recombine away from the unidentified genetic incompatibilities, our simulations showed that most markers were likely surrounded by multiple such incompatibility factors within each introgressed segment.

Our results come closest to hybrid zone studies that estimate the number of genes involved in hybrid fitness problems by using spatial clinal information in the zone of contact (see [33]). Results from hybrids zones agree with our experimental observations that least several hundred fitness-related genes may differentiate genomes of even incipient races or recently diverged sibling species [33],[35],[36]. Similarly, a recent study of *Rhagoletis* host races [15] suggests that a significant amount of the genome is experiencing divergent selection under natural field conditions, consistent with our experimental results. Finally, this work appears to be also largely consistent with studies that measure various aspects of hybrid fitness under natural conditions [34], [43], [44], [45]. These studies have recently documented that hybrid fitness compared to parental individuals is particularly affected by competitive conditions [34]. Both our experimental work and these studies are suggesting that we may be fundamentally underestimating the extent of fitness divergence that lead to incompatibilities between incipient and sibling species.

### Why are genetic incompatibilities extensive at this early stage of speciation?

Adaptive evolution within species largely rests on the basic parameters of genetic architecture of fitness-related traits [3], [46], [47], [48], [49], [50]. Such parameters as the level of genetic interactions (epistasis), the number of genes and their effects and the pleiotropic byproduct of genes will determine how much fitness and functional divergence is expected between species. If most phenotypes and developmental systems are governed by complex genetic architectures, whose genes are organized into epistatic networks that also have pleiotropic effects, we would expect that even incipient species would exhibit a multitude of fitness and functional differences between their genomes that cannot be easily broken down by subsequent gene flow [3], [6], [18], [51], [52]. This highly co-adaptive view of speciation was strongly favored by E. Mayr who even suggested that speciation will sometimes lead to veritable “genetic revolutions” due to the large-scale reorganization of allelic selective pressures as a result of new independent mutations and a change in epistatic interactions between new and existing alleles in each isolated population [3]. However, if most fitness-related traits and developmental systems are governed by few loci of additive and non-pleiotropic major effect, then it is conceivable that incipient speciation would only involve a handful of divergent loci with the rest of the genome being highly penetrable to gene flow [6]. The fact that we observed genetic incompatibilities with every random genetic introgression from *D. sechellia* into *D. simulans* suggests that the genetic basis of speciation is likely to be highly polygenic and epistatic between these young species.

### What explains the observed fitness inferiority of introgressed regions?

Our competition results are particularly striking because we showed that while these introgressions are viable and fertile on their own, they nevertheless rapidly decline in frequency when they compete against wild-type alleles for multiple generations. We studied two obvious components of fitness that could have been potentially involved in the inferiority of *D. sechellia* introgressions. These included both premating (mating success) and postmating (fertility) assays in our introgression lines (*D. simulans* background+*D. sechellia* introgressed segment) relative to the experimental *D. simulans* strain. Our results did not detect significant fertility effects of introgression since we initially showed that fertility is not lower in the introgression lines compared to the *D. simulans* strain. This finding indicates that the observed competitive exclusion of *D. sechellia* introgressions is unlikely a result of “weak” sterility and/or inviability factors since these would have generated lower fertility in introgression lines. Therefore, the cause of *D. sechellia* introgression inferiority is likely to be in other components of fitness.

We also used multiple-choice mating trials to assess relative mating success of introgression lines against *D. simulans* strain. While individuals from introgression lines had a tendency to have lower mating success compared to the *D. simulans* line, this trend was not significant. Moreover, we could not control for inbreeding effects on mating success with this approach. Taken together, these assays could not identify a clear mechanism by which *D. sechellia* alleles were outcompeted from the *D. simulans* genetic background in our experiments. At this point we can only speculate that other as of yet unknown aspects of fitness particularly involved in soft-selection or competitive ability must be responsible for these fitness incompatibilities between these genomes.

### Will more incipient and sympatric cases support the “genic view” of speciation?

What is presently unclear is which biogeographical conditions of speciation will facilitate the rapid accumulation of genetic incompatibilities. In our work we have shown that fitness incompatibilities are fairly extensive between 250,000 year old allopatric sibling species. Because these species most likely diverged in allopatry, their genomes are expected to have accumulated incompatibilities at more or less homogeneous rates over time without much gene flow [6]. Will younger sibling species also show similar patterns? Will parapatric or sympatric modes of speciation favor a more limited accumulation of genetic incompatibilities than what we have observed? While earlier studies of sequence divergence using small number of markers generally found “genomic islands of speciation” (e.g. [9], [11]), more recent analyses of incipient parapatric and sympatric forms show more extensive sequence differentiation [15]–[17]. However, it is still largely unknown whether any of these sequence differences will translate to fitness divergence and genetic incompatibilities (but see [15]).

Future work will gain further insights into the evolution of genetic incompatibilities by extending our genetic competition experiments to even more incipient cases of speciation and those that have likely speciated with gene flow. This appears to be a more accurate way to assess which view of speciation is likely to be correct. It will also determine under which circumstances extensive genetic incompatibilities accumulate between two genomes. Follow-up studies may also reveal the causes of non-sterility and non-inviability genetic incompatibilities that are likely to be observed in such long-term competition experiments.

## Materials and Methods

### Introgression lines

The recombinant introgression lines (RILs) were kindly provided by Stuart J. Macdonald, Isabel Colson and David B. Goldstein (Oxford University). The construction and genotype checking of these introgression lines are briefly described here. *D. simulans* females from the “sim132” (European *Drosophila* Stock Centre, Umeå) line were crossed to *D. sechellia* males from the “sec S9” (Mid-America *Drosophila* Stock Center), and the resultant F_1_ females were backcrossed to *D. simulans* males. The subsequent F_2_ males were individually crossed to either three *simulans* females (P cross, P) or three F_1_ females (H cross, H) and further made homozygous by single-pair sib-mating for 18 generations (SJ Macdonald, pers. comm.). Figure S1 illustrates the genotype for each introgression lines based on the information provided by SJ Macdonald.

In total, 41 microsatellite markers, i.e., 8, 16, and 17 markers on the X, 2nd and 3rd chromosomes respectively, with an average interval of about 8 cM [53] are used in the initial genotyping. There are much fewer introgression fragments with smaller sizes on the X chromosome compared to the two autosomes (SJ Macdonald, pers. Comm.). Only 3 of the 12 lines (6H, 16H, and 94P) carry a small X chromosomal introgression (Figure S1). We therefore focused on the two autosomes for the competition experiments.

Before all experiments, we genotyped these 12 introgression lines by using one microsatellite marker per introgressed segment and found 9 lines (6H, 12H, 25H, 28H, 60H, 29P, 62P, 78P, and 94P) showed the expected *sechellia* alleles (these lines are referred to as “confirmed lines”; see Figure S1 for specific location of each marker in each confirmed line). The other three lines (16H, 37P, and 129P) showed no evidence of *sechellia* alleles at the genotyped locus (Figure S1). Nevertheless, these lines may still carry some parts of the *sechellia* introgression that could not be assessed by our genotyping. Therefore we will refer to the latter three lines as “unconfirmed lines”.

### Fertility assay of introgression lines

To see if these introgressions had any obvious viability and/or sterility effects, we assayed the overall fertility of each introgression line relative to the original wild-type *D. simulans* line without introgressions. This was done by measuring the number of offspring produced by each introgression line and comparing it to the fertility of the *D. simulans* strain. All fertility assays were performed by setting up 10 replicates of three pairs of males and females in small vials for each tested line. We allowed the mating pairs to lay eggs for 15 days, at which point all adults were cleared. We then counted the number of F_1_ progeny to determine fertility. To test for significance, we first confirmed that the fertility data did not significantly deviate from normal distribution using a Goodness of Fit test (Shapiro-Wilk test: *W* = 0.987, *p* = 0.8666). We then analyzed the whole dataset using a one-way ANOVA. To determine which specific introgression lines were significantly different from each other and from the wild-type *simulans* strain, we used a Tukey-Kramer HSD test that takes into account multiple testing. All tests were performed in JMP software (SAS).

### Establishing six populations for fitness competition experiments

To determine if there were any other fitness effects of *sechellia* alleles in a *simulans* genetic background, we performed a multi-generational competition experiment lasting twenty generations. We set up six independent competition crosses between different introgression lines: (A) 37P×25H, (B) 78P×16H, (C) 6H×129P, (D) 62P×29P, (E) 94P×28H and (F) 12H×60H. Combining the introgression lines together allowed us to control for any non-intentional effects of the introgression procedure (i.e. all lines entering the competition experiment went through the same introgression procedure). The detailed procedures of the cross are as follows: (62P×29P as an example): 50 virgin females of 62P were crossed to 50 males of 29P and 50 virgin females of 29P were crossed to 50 males of 62P. The resultant F_1_ progeny of the two bottles were mixed and allowed to lay eggs to produce a large number of F_2_ progeny, which were transferred to five bottle replicates. There were approximately 300–500 flies in each bottle. The flies were allowed to lay eggs for 4 days and then collected in 100% ethanol. For the next generation, when enough flies (300–500) emerged, they were transferred to a new bottle. The same procedure applied to other crosses. In total, we set up five replicates for each one of the six distinct populations. The population sizes were kept at 300–500 for each replicate. All experiments were done at 22±1°C with a 12 hr–12 hr light–dark cycle.

### Measuring allele frequency of the six populations

Samples of around 40 flies were taken from each bottle at generations 6 or 7, 14, and 20. For each introgression segment, we examined one microsatellite marker in that region. The microsatellite markers are: A: DMU25686 (cytological position: 93F14); B: DRODORSAL (36C8); C: DROGPAD (47A9); D1: AC005732 (cytological position 24C9); D2: DMRHO (62A2); E1: DMMP20 (49F13); E2: DMCATHPO (75E1); F1: DS00361 (54B5); F2: DMU43090 (99D5) [53], [54]. From the genotyping results, allele frequencies were calculated for each bottle replicate and for whole experiment sets.

### Maximum likelihood estimates of the selection coefficients

We developed an individual-based computer simulation model of our competition experiments performed above. The purpose of the simulation was to manipulate the presumed selection pressures against *sechellia* alleles relative to *simulans* alleles, the dominance of *sechellia* alleles' fitness effects and the recombination rate between selected loci and marker loci. The goal was to determine which combination of selection pressures and recombination rates was best at explaining our observed results.

The simulations tracked either *D. simulans* or *D. sechellia* alleles at both the marker loci and the selected loci for each individual in the population. Each simulation involved 10,000 iterations with fixed values of selection coefficient (*s*), recombination distance (*c*), and dominance (*h*). Each iteration ran for 20 generations with population sizes fixed at 150 males and 150 females each generation. Each generation was divided into the following stages: selection, recombination (females only), and reproduction. In the selection stage: individuals homozygous for the *D. simulans* allele all survived, heterozygous individuals survived with probability = *hs*, and individuals homozygous for the *D. sechellia* alleles survived with probability = *s*. Reproduction began with recombination in females which occurred with probability = *c*. During random mating, male and female haplotypes were randomly selected from the population to make 150 males and 150 females for the next generation.

Sampling took place in generations 7, 14, and 20 where 20 males and females were removed from the population after selection and before random mating. The allele frequencies of the *D. simulans* alleles at the marker loci were recorded from these sampled flies. The simulation ran for 10,000 iterations. Distributions were created for each of the three sampled generations (7, 14, and 20). The allele frequencies were assigned to one of 40 bins (0–.025, .025–.05, …, .975–1), and bin counts were incremented for each iteration. Observed allele frequencies for each marker were then compared to the three distributions. For a given generation, all of the bins containing observed frequencies were added together and divided by the total number of iterations. The log of this ratio was treated as a likelihood estimate for the parameters *s*, *c*, and *h* for that marker. Simulations were run for all combinations of *c* = 0.0001, 0.001, 0.01, 0.1, 0.2, 0.3, 0.4, and 0.5; *s* = 0.1, 0.2, 0.3, 0.4, 0.5, 0.6, 0.7, 0.8, and 0.9; and *h* = 0, 0.5, 0.9, and 1. The Maximum likelihood estimate for each marker was the set of *s*, *c*, and *h* that yielded the highest likelihood value.

### Linkage disequilibrium analyses

Linkage disequilibrium (LD) analyses were carried out for double introgression experiments (experiments D, E, and F in Figure 1). The null hypothesis is that genotypes at one locus assort independently from genotypes at the other locus. The exact test for the LD was performed by using M. Raymond and F. Rousset's GENEPOP software package (http://genepop.curtin.edu.au/genepop_op2.html). We performed this probability test using a Markov chain with parameters of dememorization number = 1000, number of batches = 100 and number of iterations per batch = 1000.

### Mating behavior assays of introgression lines in competition with the wild-type strain

To assay whether introgressed segments caused a reduction in the mating success of their individuals relative to wild-type *D. simulans* genotype, we applied a multiple-choice mating experiment design similar to [55]. All flies were fed red or green colored food 14–18 hours prior to the experiment. The food coloration alternated between replications and had no effect on *D. simulans* mating choice (data not shown). Experiments were started within one hour after the beginning of the light cycle and conducted at 22±1°C. Sixty 4-day-old virgin flies from each sex of *D. simulans* S132 line and the introgression line were simultaneously released into a Plexiglas cage (14.5″ L×10″ H×9.5″ W) with fly food in a 14-cm diameter Petri dish. The copulating pairs were aspirated out of the cage for identification by the food coloring in the guts. We let the mating trials run for 1 hour or until 60 matings (50% of possible copulations) had occurred, whichever came first (as recommended by [56] to avoid bias). Several comparisons were replicated at least three times to determine overall reliability in the mating behavior. We then calculated the percentage of matings by *D. simulans* individuals relative to introgression line individuals and also the relative percentage of matings by each sex of each line.

## Supporting Information

Figure S1 Cytological positions of the recombinant introgression lines (SJ Macdonald, pers. comm.) chosen in establishing initial generations (G1) of six populations (A–F) for fitness competition experiment. The light bars represent *simulans* chromosomes, and the dark blocks represent *sechellia* introgression regions. The grey blocks represent the regions not showing expected *sechellia* microsatellite markers DROGPDHA (26A3)+AC005555 (29A4), DM22F11T (73A2), and DMU43090 (99D5) in the lines 37P, 16H, and 129P, respectively. The black-and-white stripes are used to indicate the boundary of the introgressed segment lying somewhere between the adjacent microsatellite markers which exhibit different species patterns. The cytological position of each marker is indicated by a vertical line or a reverse triangle on the second (cytological region: 21–60) and third (61–80) chromosomes based on the map of *D. melanogaster*. The long arrow bar on the third chromosome indicates the large inverted region (84F6-7–93F6-7) compared to *D. melanogaster*. One microsatellite marker tracked for each introgression in the competition experiments is indicated by a reverse triangle. The microsatellite markers tracked are: A: DMU25686 (cytological position: 93F14); B: DRODORSAL (36C8); C: DROGPAD (47A9); D1: AC005732 (cytological position 24C9); D2: DMRHO (62A2); E1: DMMP20 (49F13); E2: DMCATHPO (75E1); F1: DS00361 (54B5); F2: DMU43090 (99D5) [53], [54].
(PDF)

Figure S2 The contour plots of the maximum likelihood estimates for each microsatellite marker used in Table 1. Vertical axis represents recombination rate (*c*) and horizontal axis represents selection coefficient (*s*) for all plots. Dominance of the *sechellia* allele (*h*) ranges from 0, 0.5, 0.9 to 1 and from left to right for each marker contour plots (see above each plot). The red X mark in each contour plot represents the maximum likelihood estimate for the specific marker (summarized in Table 1).
(DOC)

Table S1 Linkage disequilibrium tests between 2^nd^ and 3^rd^ chromosome microsatellite markers in double introgression experiments.
(DOC)

Table S2 Fertility of parental individuals of each introgression line outcrossed to a different introgression line and the fertility of their F_1_ progeny.
(DOC)

Table S3 Fertility of unconfirmed lines in comparison to *D. simulans* strain.
(DOC)
