# Supplementary figures and images for: Incompatibility and Competitive Exclusion of Genomic Segments between Sibling Drosophila Species
Source: PLoS Genet. 2012 Jun 28;8(6):e1002795. doi: 10.1371/journal.pgen.1002795 (PMC3386244; doi:10.1371/journal.pgen.1002795)

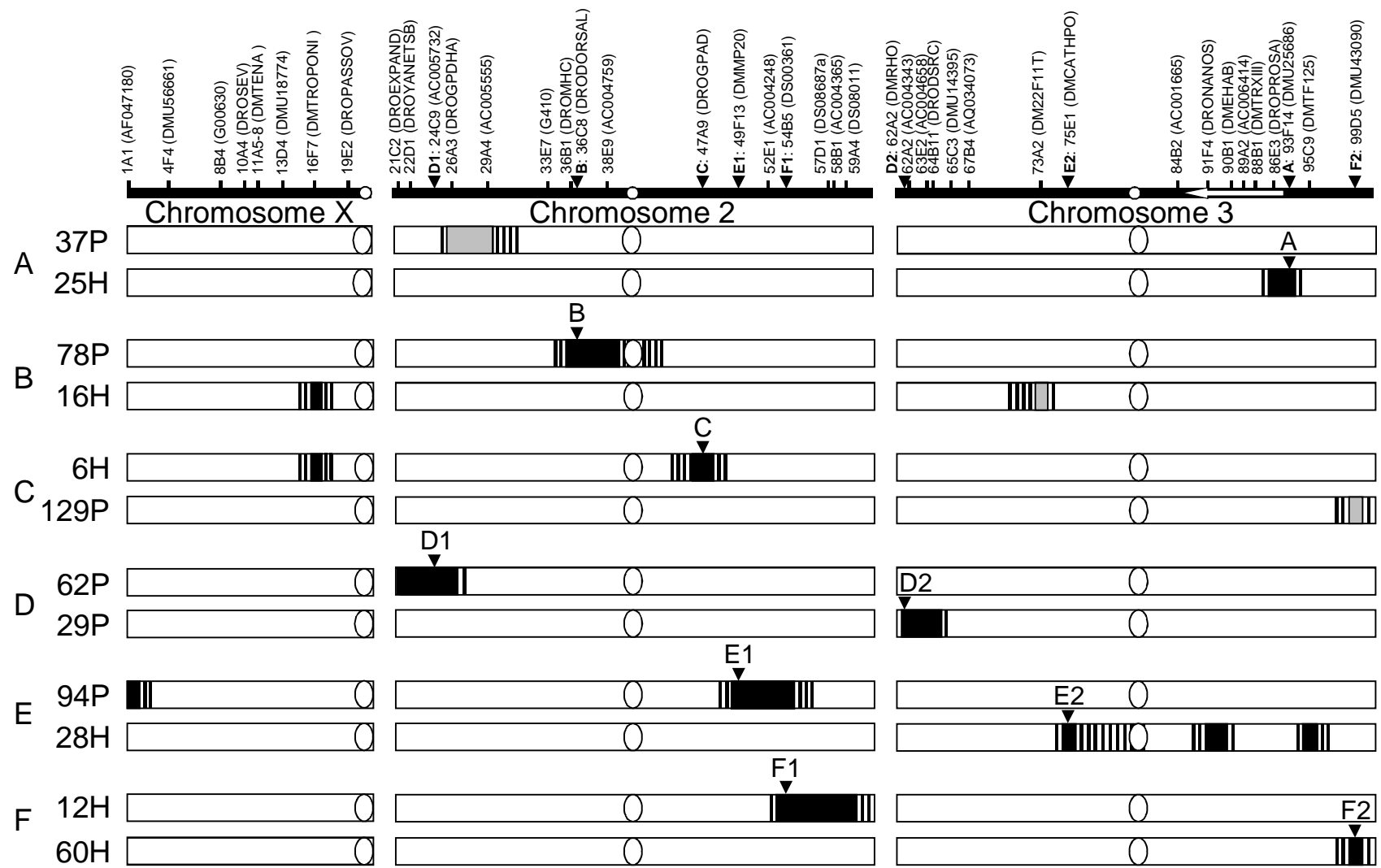

Supplement: Figure S1 — Cytological positions of the recombinant introgression lines (SJ Macdonald, pers. comm.) chosen in establishing initial generations (G1) of six populations (A–F) for fitness competition experiment. The light bars represent simulans chromosomes, and the dark blocks represent sechellia introgression regions. The grey blocks represent the regions not showing expected sechellia microsatellite markers DROGPDHA (26A3)+AC005555 (29A4), DM22F11T (73A2), and DMU43090 (99D5) in the lines 37P, 16H, and 129P, respectively. The black-and-white stripes are used to indicate the boundary of the introgressed segment lying somewhere between the adjacent microsatellite markers which exhibit different species patterns. The cytological position of each marker is indicated by a vertical line or a reverse triangle on the second (cytological region: 21–60) and third (61–80) chromosomes based on the map of D. melanogaster. The long arrow bar on the third chromosome indicates the large inverted region (84F6-7–93F6-7) compared to D. melanogaster. One microsatellite marker tracked for each introgression in the competition experiments is indicated by a reverse triangle. The microsatellite markers tracked are: A: DMU25686 (cytological position: 93F14); B: DRODORSAL (36C8); C: DROGPAD (47A9); D1: AC005732 (cytological position 24C9); D2: DMRHO (62A2); E1: DMMP20 (49F13); E2: DMCATHPO (75E1); F1: DS00361 (54B5); F2: DMU43090 (99D5) [53], [54]. (PDF) [file pgen.1002795.s001.pdf]

A

B

C


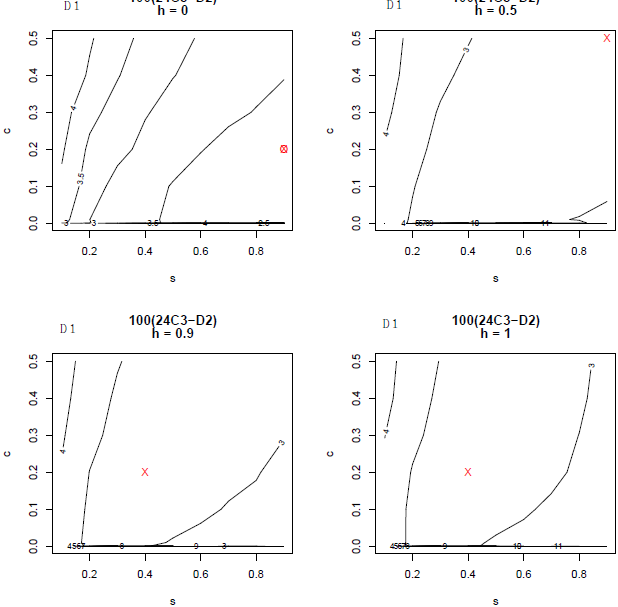


D1

D2

E1

E2

F1

F22

Supplement: Figure S2 — The contour plots of the maximum likelihood estimates for each microsatellite marker used in Table 1. Vertical axis represents recombination rate (c) and horizontal axis represents selection coefficient (s) for all plots. Dominance of the sechellia allele (h) ranges from 0, 0.5, 0.9 to 1 and from left to right for each marker contour plots (see above each plot). The red X mark in each contour plot represents the maximum likelihood estimate for the specific marker (summarized in Table 1). (DOC) [file pgen.1002795.s002.doc]
