# Supplementary material for: Incompatibility and Competitive Exclusion of Genomic Segments between Sibling Drosophila Species
Source: PLoS Genet. 2012 Jun 28;8(6):e1002795. doi: 10.1371/journal.pgen.1002795 (PMC3386244; doi:10.1371/journal.pgen.1002795)
Supplement: Table S1 — Linkage disequilibrium tests between 2nd and 3rd chromosome microsatellite markers in double introgression experiments. (DOC) [file pgen.1002795.s003.doc]

| Microsatellite | Competition |  | G7 |  |  | G14 |  |  | G20 |  |
| --- | --- | --- | --- | --- | --- | --- | --- | --- | --- | --- |
| Markers | experiments | | P value | ± S. E. |  | P value | ± S. E. |  | P value | ± S. E. |
| D1 & D2 |  |  |  |  |  |  |  |  |  |  |
|  | D-1 |  | 1.0000 | ± 0.0000 |  | 0.5022 | ± 0.0071 |  | 0.0375 | ± 0.0019* |
|  | D-2 |  | 0.3298 | ± 0.0063 |  | 0.7321 | ± .00415 |  | 0.0320 | ± 0.0019* |
|  | D-3 |  | 0.0124 | ± 0.0016* |  | 1.0000 | ± 0.0000 |  | 0.8435 | ± 0.0028 |
|  | D-4 |  | 0.9373 | ± 0.0018 |  | 0.0041 | ± 0.0005** | | 0.1382 | ± 0.0036 |
|  | D-5 |  | 0.8161 | ± 0.0032 |  | 0.4271 | ± 0.0051 |  | 0.2873 | ± 0.0041 |
| E1 & E2 |  |  |  |  |  |  |  |  |  |  |
|  | E-1 |  | ND |  |  | 1.0000 | ± 0.0000 |  | NA |  |
|  | E-2 |  | ND |  |  | 0.5120 | ± 0.0032 |  | 0.2548 | ± 0.0039 |
|  | E-3 |  | 0.8073 | ± 0.0038 |  | 0.1319 | ± 0.0025 |  | 0.1559 | ± 0.0048 |
|  | E-4 |  | 0.5556 | ± 0.0058 |  | 1.0000 | ± 0.0000 |  | 0.1564 | ± 0.0019 |
|  | E-5 |  | 0.8647 | ± 0.0017 |  | 1.0000 | ± 0.0000 |  | 0.4029 | ± 0.0026 |
| F1 & F2 |  |  |  |  |  |  |  |  |  |  |
|  | F-1 |  | 0.0944 | ± 0.0025 |  | 0.7667 | ± 0.0029 |  | 1.0000 | ± 0.0000 |
|  | F-2 |  | 1.0000 | ± 0.0000 |  | 1.0000 | ± 0.0000 |  | NA |  |
|  | F-3 |  | 0.3153 | ± 0.0040 |  | 0.5326 | ± 0.0038 |  | 0.2518 | ± 0.0033 |
|  | F-4 |  | 1.0000 | ± 0.0000 |  | 1.0000 | ± 0.0000 |  | 1.0000 | ± 0.0000 |
|  | F-5 |  | 0.1826 | ± 0.0027 |  | 1.0000 | ± 0.0000 |  | 0.7611 | ± 0.0040 |

Note: ND = not done; NA = not available due to fixation of the *simulans* allele; Exact test performed by using M. Raymond and F. Rousset’s GENEPOP software package (<http://genepop.curtin.edu.au/genepop_op2.html>): * *p* < 0.05, ** *p* < 0.01
