# Supplementary material for: Incompatibility and Competitive Exclusion of Genomic Segments between Sibling Drosophila Species
Source: PLoS Genet. 2012 Jun 28;8(6):e1002795. doi: 10.1371/journal.pgen.1002795 (PMC3386244; doi:10.1371/journal.pgen.1002795)
Supplement: Table S2 — Fertility of parental individuals of each introgression line outcrossed to a different introgression line and the fertility of their F1 progeny. (DOC) [file pgen.1002795.s004.doc]

| Crosses (♀ x ♂) | Parental fertility Mean ± SE  (N = 7) | F_1_ fertility  Mean ± SE  (N = 7) |
| --- | --- | --- |
| B (78P x 16H) | 425 ± 11 | 465 ± 44 |
| B (16H x 78P) | 434 ± 52 | 497 ± 39 |
| C (6H x 129P) | 233 ± 47 | 457 ± 42 |
| C (129P x 6H) | 476 ± 75 | 486 ± 82 |
| D (62P x 29P) | 488 ± 31 | 485 ± 76 |
| D (29P x 62P) | 382 ± 41 | 478 ± 85 |
| E (94P x 28H) | 235 ± 62 | 425 ± 71 |
| E (28H x 94P) | 288 ± 54 | 425 ± 50 |
| F (12H x 60H) | 311 ± 115 | 543 ± 52 |
| F (60H x 12H) | 307 ± 37 | 547 ± 42 |

Notes: Methods of fertility design and measurement are identical to that of Table 1. Wilcoxon/Kruskal-Wallis Tests: Mean Fertility: Parental crosses = 353(SE = 13), F1 crosses = 486 (SE = 8.50), *Simulans123* strain = 273(SE = 27), Z-value _Parental vs_. *_Simulans_* = -1.65, *P*-value = 0.10; Z-value _F1 vs_. *_Simulans_* = 4.83, *P*-value < 0.0001. Significantly higher fertility between F1 progeny likely suggests that the lower fertility of introgression lines and *simulans* line is due to inbreeding effects.
