# Supplementary material for: Incompatibility and Competitive Exclusion of Genomic Segments between Sibling Drosophila Species
Source: PLoS Genet. 2012 Jun 28;8(6):e1002795. doi: 10.1371/journal.pgen.1002795 (PMC3386244; doi:10.1371/journal.pgen.1002795)
Supplement: Table S3 — Fertility of unconfirmed lines in comparison to D. simulans strain. (DOC) [file pgen.1002795.s005.doc]

| Lines | Progeny Mean ± SE | Tukey-Kramer HSD test value* | Significance |
| --- | --- | --- | --- |
| sim 132 (*D. simulans*) | 289 ± 82.4 |  | NS |
| undetermined lines (average) | 343 |  |  |
| 37P (A) | 239 ± 69.8 | -47.594 | NS |
| 16H (B) | 401 ± 78.1 | 13.606 | < 0.05 |
| 129P (C) | 389 ± 58.6 | 1.506 | < 0.05 |

Notes: Methods of fertility design and measurement are identical to that of Table 1.
